# Partial substitution of alfalfa hay by *Berberis vulgaris* leaf modulated the growth performance, meat quality and antioxidant status of fattening lambs

**DOI:** 10.1002/vms3.934

**Published:** 2022-09-16

**Authors:** Seyed Morteza Vaghar Seyedin, Mohsen Mojtahedi, Seyed Homayoun Farhangfar, Navid Ghavipanje

**Affiliations:** ^1^ Department of Animal Science Faculty of Agriculture University of Birjand Birjand Iran

**Keywords:** antioxidants, *Berberis vulgaris*, fattening lamb, meat quality

## Abstract

**Background:**

Undoubtedly, global warming has caused a decrease in the production of agricultural commodities. This problem has increased the price of animal feed due to competition with human consumption. Meanwhile, the physiology of ruminants gives them the ability to use by‐products and agricultural waste and supply their requirements for growth, maintenance and even production. *Berberis vulgaris* is a plant native to Iran, and after separating the fruit, its waste (mainly leaf) is unused and causes environmental pollution. The leaves of this plant contain significant amounts of phenolic compounds, alkaloids and anthocyanins that have antioxidant properties.

**Objectives:**

This experiment was conducted with the aim of determining the chemical properties of barberry leaves, such as crude protein, phenolic compounds, tannins and alkaloids. The effects of substituting of *B. vulgaris* leaf (BVL) in the diet on performance characteristics of fattening Baluchi lambs were evaluated. The quality and antioxidant status of meat and blood parameters such as glucose, cholesterol, blood urea nitrogen and liver enzymes were investigated.

**Material and methods:**

A total of 21 male of 5–6 months old lambs with a mean body weight of 30.60 ± 1.28 kg were randomly assigned to one of three dietary treatments with different levels of BVL: 1–diet without BVL (control), 2–diet containing 7.5% BVL (BVL7.5), and 3–diet containing 15% BVL (BVL15; dry matter [DM] basis). Blood samples were harvested after overnight fasting from the jugular vein at 0, 28, 56 and 84 days. The lambs were slaughtered after 84 days of feeding trial and longissimus dorsi (LD) muscle was dissected. Meat quality and antioxidant stability status were measured.

**Results:**

15% substitution of alfalfa hay by BVL (BVL15) increased DM intake) and decreased average daily gain (*p* ≤ 0.05). The LD muscle (*p* ≤ 0.05), liver (*p* ≤ 0.01) and plasma (*p* ≤ 0.05) samples of lambs fed either BVL7.5 or BVL15 displayed a greater total antioxidant capacity than that of lambs fed the control diet. Also, malondialdehyde concentration was decreased in plasma (*p* ≤ 0.01) and LD muscle of lambs (*p* ≤ 0.05) fed both BVL7.5 and BVL15. In addition, higher a* and C* values (*p* ≤ 0.05) were observed in the meat of lambs fed BVL15 than those fed with the control, while the lightness (L*) in BVL15 was lower, compared to other experimental diets

**Conclusions:**

Overall, our results indicated that 7.5% substitution of alfalfa hay by BVL may positively modulate the antioxidant status of fattening lambs and improve the colour stability of meat without negative effects on performance characteristics.

## Graphical abstract

For the first time, a comparison is made between the chemical composition of alfalfa hay and *Berberis vulgaris* leaf (BVL). The growth performance of lambs fed 7.5% BVL were the same as that of alfalfa hay. Feeding BVL improved the quality, total antioxidant capacity and colour stability of lamb meat, compared to alfalfa.

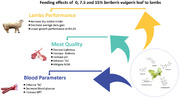

## 1 INTRODUCTION

The role of ruminants, especially small ruminants, in providing livelihood and even the advantage of many human populations in developing countries is not hidden from anyone (Ahsani et al., 2010). These livestocks are less affected by artificial breeding programmes and often have low production efficiency, compared to their commercial meat and dairy breeds (Mohammadabadi et al., 2018). However, the resistance of these in harsh weather conditions, the use of poor feed resources and higher resistance to diseases have caused them to be widely used by small‐scale farmers. On the other hand, by 2050, the global demand for meat is projected to increase by 58%, which will turn the sustainable meeting of the global food supply into a major challenge of the 21st century (Makkar et al., 2014). Lamb meat is one of the important sources of animal protein with a balanced fatty acid profile and no limiting amino acids (Elmore et al., 2005; Krishtafovich et al., 2016). However, it is a relatively perishable product with a shelf life of 7−10 days (Camo et al., 2008; Karabagias et al., 2011) since its high moisture content, water activity (aw) and pH values allow microbial spoilage (Osés et al., 2013) and foodborne pathogens growth (Lenahan et al., 2007; Sierra et al., 1995). Lipid oxidation of meat during post‐mortem ageing has been related to its deterioration of flavour, colour, odour, quality and nutritive value (Luciano et al., 2009), which can be reduced by dietary supplementation of antioxidants (Coronado et al., 2002).

Nowadays, concerning meat purchases, consumers are increasingly using extrinsic cues to perceive quality, which is associated with nutrition and health (da Fonseca & Salay, 2008; Grunert, 2006). Hence, many synthetic and natural substances have been investigated as potential antioxidants to prevent lipid oxidation. The trend is to decrease the use of synthetic antioxidants due to consumer concerns over safety and toxicity (Coronado et al., 2002). As a result, the search for natural antioxidants, especially of plant origin, has notably increased in recent years.

*Berberis vulgaris* belongs to the *Berberidaceae* family and is a small shrub with yellow to brown‐coloured bark. The plant has obviated leaves, bearing pendulous yellow flowers in spring succeeded by oblong red‐coloured fruits (barberry; Rahimi‐Madiseh et al., 2017). Iran is the biggest producer of this edible fruit, and currently, there is over 11,000 ha of cultivated lands that produce more than 9200 tons of dried fruit per year (Alemardan et al., 2013). Various parts of this plant, including its root, bark, leaf and fruit have been already used in folk medicine worldwide (Imanshahidi & Hosseinzadeh, 2008). Different compounds, including alkaloids, flavonoids, sterols, vitamins and carotenoids have been identified in this plant (Karimov, 1993). Research in the last decade shows the anti‐bacterial (Akbulut et al., 2009; Imanshahidi & Hosseinzadeh, 2008), anti‐carcinogenic (Bhatt, 2016), anti‐histaminic (Shamsa et al., 1999), anti‐hyperglycemic (Meliani et al., 2011), antioxidant (Murugesh et al., 2005), anti‐inflammatory (Mokhber‐Dezfuli et al., 2014), anti‐hypertensive (Saki et al., 2016) and lipid‐lowering (Rahimi‐Madiseh et al., 2017) effects of *B. vulgaris* leaf (BVL).

The radical scavenging activity of BVL alkaloids has also been well‐established (M. H. Jang et al., 2009). It had been demonstrated that BVL could enhance the activity of enzymatic antioxidants including superoxide dismutase, glutathione peroxidase and catalase as well as reduce lipid oxidation rate in rats (Laamech et al., 2017; Wu et al., 2015; Yang et al., 2017). Feeding BVL to lactating goats (at levels 17.5% and 34% dry matter [DM]) not only had no adverse effects on milk production, milk composition and body weight gain (BWG) but also increased the total antioxidant capacity (TAC) as well as DM intake (DMI) and milk fat content (Ghavipanje et al., 2016). Also, phytoestrogens, which are structurally similar to estrogen, have been identified in plants that contain lignans, flavonoids (such as isoflavonoids), lactones, silicic acid residues and comets (Masoudzadeh et al., 2020). Among the most important effects of these compounds, we can mention the improvement of blood sugar control, reduction of blood cholesterol and decrease of inflammation without negative effect on male fertility (Ceccarelli et al., 2021). In fact, the easy decomposition of these compounds causes them to remain in the body for a short time and not be stored in the tissues. Therefore, these phytoestrogens are likely to be safe and beneficial if consumed as part of a regular diet in the short term (Shahsavari et al., 2021). Therefore, it seems that BVL can act as a natural antioxidant owing to its high levels of bioactive compounds such as phenols and alkaloids. However, as far as we know, no study has been conducted to determine the effect of BVL on meat oxidative stability and carcass quality of lambs. Therefore, this experiment was performed to clarify the effects of partial substitution of alfalfa hay by BVL on the growth performance, meat quality and antioxidant status of fattening lambs.

## 2 MATERIALS AND METHODS

### 2.1 Animals, diets and experimental design

This experimental study was performed following the guidelines of the Iranian Council of Animal Care (protocol ID 19,293). The current study was conducted on the experimental farm of the Faculty of Agriculture, University of Birjand, Birjand, Iran (longitude and latitude, 37.42° N and 57.31° E).

Twenty‐one Baluchi male lambs, 5–6 months old with an average initial body weight (BW) of 30.60 ± 1.28 kg were randomly assigned to one of three dietary treatments (*n* = 7 per group). BVL used in this study was prepared from a barberry processing factory in Birjand, South Khorasan, Iran. At the beginning of the trial, all the animals were treated for external and internal parasites and vaccinated against enterotoxemia (Enteroprotect P 100, Razi Vaccine and Serum Research Institute). The dietary treatments included different levels of BVL as follows: 1–diet without BVL (control), 2–diet containing 7.5% BVL (BVL7.5), and 3–diet containing 15% BVL (BVL15; DM basis). Diets were formulated to be isocaloric and isonitrogenous, according to National Research Council (2007) requirements (Table 1). The experiment lasted 84 days following an adaptation period of 14 days. Lambs were housed individually in slatted floor pens (1.5 × 2 m). The feed was offered as total mixed ration twice daily at 07:00 AM and 05:00 PM, and the amounts of diet fed and refusals were weighed daily for each lamb to determine dry matter intake (DMI). The quantity of feed offered was adjusted daily with a 20% excess of the daily intakes to ensure ad libitum consumption. The lambs were weighed individually before the morning feeding on Days 0, 28, 56 and 84 to determine the average daily gain (ADG) and feed conversion ratio (FCR; DMI [g/day]/ADG [g/day]).

**TABLE 1:** Ingredient and chemical composition of experimental diets (% DM)

	Experimental diets^a^		
	Control	BVL7.5	BVL15
**Ingredient (% of dry matter [DM])**			
Alfalfa hay	30	22.5	15
*Berberis vulgaris* leaf^b^ (BVL)	0	7.5	15
Corn ground	16	16	16
Barley ground	34	34	34
Cottonseed meal	5	5	5
Soybean meal	3	3	3
Wheat bran	5	5	5
Beet molasses	4	4	4
Sodium bicarbonate	0.5	0.5	0.5
Calcium carbonate	1	1	1
Salt	0.5	0.5	0.5
Vitamin‐mineral supplement^c^	1	1	1
**Chemical composition of diet (% of DM)**			
Metabolizable energy	2.41	2.43	2.46
Ether extract	2.11	2.18	2.25
Crude protein (CP)	14.35	14.30	14.26
Ash	6.94	6.89	6.82
Neutral detergent fibre (NDF)	27.92	27.43	26.95
Acid detergent fibre (ADF)	17.10	16.01	14.93
Non‐fibre carbohydrates	48.68	49.20	49.72

^a^ Control, BVL7.5 and BVL15 contained 0%, 7.5% and 15% BVL (DM basis), respectively.

^b^ Contains 95.5% DM, 13.80% CP, 2.13% ether extract, 7.88% ash, 34.27% NDF, 17.97% ADF (DM basis).

^c^ Containing vitamin A (250,000 IU/kg), vitamin D (50,000 IU/kg) and vitamin E (15,000 IU/kg), manganese (2.25 g/kg), calcium (120 g/kg), zinc (7.7 g/kg), phosphorus (20 g/kg), magnesium (20.5 g/kg), sodium (18.6 g/kg), iron (1.25 g/kg), sulfur (3 g/kg), copper (1.25 g/kg), cobalt (14 mg/kg), iodine (56 mg/kg) and selenium (10 mg/kg).

### 2.2 Blood collection, slaughter procedures and muscle sampling

Blood samples (10 ml/lamb) were harvested after overnight fasting from the jugular vein using heparinised blood collection tubes (RotexMedica) at Days 0, 28, 56 and 84. Plasma samples were obtained by centrifuging the blood tubes for 15 min at 3000 × g and then were frozen at −80^◦^C until analysis.

On the slaughter day, lambs were overnightly fasted and their BW was recorded. Then the animals were slaughtered and immediately the weight of the hot carcass was recorded. The weights of leg and hoof, head, skin, testis, liver, lung, spleen, kidney, heart, visceral fat, kidney and heart fat, full and empty rumen and intestine were recorded after the removal of the contents. All procedures employed in this study (transport and slaughtering) meet ethical guidelines and adhere to Iranian legal requirements. The length between the anterior edge of the first rib and the anterior end of the pubic symphysis was calculated as carcass length. The weight of the shoulder, leg, loin, ribs, flank and neck was recorded according to Colomer‐Rocher et al. (1987). Thereafter, the left side of the carcass was used for measurements of meat quality parameters. Immediately after slaughtering, approximately 300 g of the left longissimus dorsi (LD) and some hepatic samples were sampled and immediately stored at 4°C for determination of thiobarbituric acid reactive substances (TBARS) values.

### 2.3 Chemical analysis

Samples of BVL were dried at 60°C, milled to pass a 1 mm screen using a Wiley mill (Retsch Cutting Mill), and were analysed for organic matter, crude protein and ether extract according to the Association of Official Analytical Chemists (AOAC) (2000). Neutral detergent fibre and acid detergent fibre were determined according to Van Soest et al. (1991). Samples of BVL were extracted by shaking at room temperature with methanol–water (80:20 vol/vol, 50 ml/g of BVL flour) for 60 min. After centrifugation (15 min, 3000 × *g*), supernatants were collected and kept in the dark at 4°C until analysis for phenolic compounds. Total phenols, flavonoids and tannins were analysed as described by Makkar et al. (1993). The anthocyanin content was also determined according to Rapisarda et al. (2000). Briefly, 2 ml of BVL extract was diluted up to 25 ml with a pH = 1 buffered solution. Then 2 ml of another aliquot was diluted up to 25 ml with a pH = 4.5 solution. The absorbance of the solutions was measured at 510 nm (Unico Spectrophotometer, 2800 UV‐visible). The absorbency difference between the two pH values was calculated as follows:

(1) $$A=\left[\left(A_{\max}-A_{700\;\mathrm{nm}}\right)\mathrm{pH}1.0\right]-\left[\left(A_{\max}-A_{700\;\mathrm{nm}}\right)\mathrm{pH}4.5\right].$$

Then, the concentration of total anthocyanin (mg/L) was calculated using the following equation:

(2) $$\mathrm{Total}\;\mathrm{Anthocyanin}\;\left(\mathrm{mg}/L\right)=\left(A\times \mathrm{MV}\times \mathrm{DF}\times 1000\right)/\left(\in \times d\right),$$

where A is absorbance, A_max_ is the absorbance at 510 nm, A_700_ is the absorbance at 700 nm, MW is the molecular weight of the pelargonidin 3‐glucoside = 433.39 g/mol, DF is the dilution factor = 10, ϵ is the coefficient of molar absorptivity = 15,600, d is the pathlength (cm) = 1.

The spectrophotometric determination of total alkaloids was performed according to Shamsa et al. (2008). Briefly, the plant materials (100 g) were extracted with methanol. The extract was filtered and a part of the residue was dissolved in 2 N HCl and then again filtered. One millilitre of this solution was transferred to a separatory funnel and washed with 10 ml chloroform (three times). Then 5 ml of bromocresol green solution and 5 ml of phosphate buffer were added to this solution. The mixture was shaken and the complex formed was extracted with 1‐, 2‐, 3‐ and 4‐ml chloroform. The extracts were collected in a 10‐ml volumetric flask and diluted to volume with chloroform. The absorbance of the complex in chloroform was measured at 470 nm (Unico Spectrophotometer, 2800 UV/visible).

### 2.4 TAC and malondialdehyde (MDA) determination

The MDA content in plasma was determined by thiobarbituric acid (TBA) reaction according to Placer et al. (1966), and the values were expressed in terms of nanomole MDA per millilitre of plasma. Lipid oxidation of ground samples of liver and muscle (24 h of storage at 4°C(was assessed by using the TBARS method described by Esterbauer and Cheeseman (1990). Results were expressed as mg of MDA/kg of liver or LD muscle.

The TAC analysis of meat and liver samples was conducted using the method of Benzie and Strain (1996). Ten grams of each meat and liver sample were homogenised in potassium phosphate buffer (pH = 7.0). Homogenates were centrifuged at 10,000 × *g* for 30 min at 4°C and the supernatant was collected. Three hundred microlitre of distilled water was mixed with 100 μl of muscle or liver extract and 3 ml of ferric reducing‐antioxidant power reagent, and the resultant mixture was incubated at 37°C for 4 min. The absorbance at 593 nm was recorded and reduction power activities were expressed as micromole equivalents of Fe^2+^. Plasma TAC was determined with commercially available Randox kits. All measurements were performed in triplicate at last and according to the manufacturer's instructions.

### 2.5 Measurement of pH and meat colour stability

Muscle pH, colour coordinates, drip loss, cooking loss and shear force were determined following the protocol described by Lokman et al. (2017). In short, to measure pH24, about 10 g of LD muscle (between the 11th and 14th ribs) was cut and mixed with 90 ml of distilled water. The mixture was then passed through Whatman filter paper (150 mm in diam.). Finally, the pH was recorded by a pH meter (Metrohm 744). Meat colour was measured 24 h after slaughter using a Hunterm lab colorimeter (HunterLab, D25, optical sensor, DP‐9000) and protocol described by L* (lightness), a* (redness) and b* (yellowness) system (CIE, 1986). Before the measurements of the L*, a* and b*, the chromameter was calibrated. Also, the following equations were used to measure the hue angle (H*) and chroma (C*):

(3) $$H*=\arctan\left(b*/a*\right),$$

(4) $$C*={\left(a{*}^{2}+b{*}^{2}\right)}^{1/2}.$$

Shear force was measured according to the method published by Hopkins and Thompson (2001) for lamb meat.

### 2.6 Statistical analysis

A completely randomised design with three treatments (diets) and seven replicates (lams) was used for this study. All the data were statistically analysed using the MIXED procedure of SAS (2002; version 9.2, SAS Institute Incorporation) for repeated measures. The fixed effects in the model were: the dietary treatment (diet), the time of sampling (time) and their interaction (diet × time), while lambs were included as a random factor. The initial BW and blood parameters of each lamb were used as covariates in the model (Equation 5). Least‐square means (LSM) were calculated and tested for differences by Tukey's test. Differences in LSM were significant at *p* ≤ 0.05, and *p* ≤ 0.10 was considered a tendency.

(5) $$Y_{ijkl}=\mu +T_{i}+Q_{j}+{\left(T\times Q\right)}_{ij}+C_{k}+e_{ijkl}.$$

In this model, *Y_ijkl_* is the dependent variable, *μ* is the average of total observations, *T_i_* is the effect of diet treatment, *C_k_* is the covariate (initial weight or blood parameters difference), *Q_j_* is the effect of sampling time, (*T* × *Q*)*_ij_* is the interaction effect of treatment and time, and *e_ijkl_* is the random error.

## 3 RESULTS

### 3.1 Chemical composition, phenolic compounds and total alkaloids of BVL

Total phenol, tannin, flavonoid and anthocyanin levels in BVL were significantly higher than in alfalfa hay (*p* < 0.0001, Table 2).

**TABLE 2:** Phenolic compounds, total alkaloid and flavonoids of BVL and alfalfa hay^a^

Item	Alfalfa hay	BVL	SEM^b^	*p*‐value
Total phenolic compound (mg/g)	12.43^b^	14.59^a^	0.0269	0.0001
Total tannin (%)	0.74^b^	5.94^a^	0.0174	0.0001
Condensed tannin (%)	0.08^b^	0.75^a^	0.0139	0.0001
Hydrolysable tannin (%)	0.65^b^	5.19^a^	0.0093	0.0001
Total alkaloid (mg/g)	ND	1.18	0.0149	–
Total flavonoid (mg QUE^c^/g)	12.84^b^	42.57^a^	0.1330	0.0001
Anthocyanin (mg CGE^d^/g)	1.02^b^	10.33^a^	0.0451	0.0001

*Note*: Values within a row with different superscripts differ significantly *(p* < 0.05).

^a^ Values are least‐square means (LSM).

^b^ SEM = pooled standard error of the mean.

^c^ QUE= Quercetin.

^d^ CGE= cyanidin‐3‐glucoside equivalents

ND = not detected.

### 3.2 DMI and growth performance

Daily feed intake and growth performance of lambs are presented in Table 3. Feeding BVL15 significantly increased DMI in lambs (*p* ≤ 0.05); however, there were no differences among BVL7.5 and control groups. The final BW (FBW) in the BVL7.5 and control groups were significantly higher than the BVL15 (*p* < 0.0001). The inclusion of 7.5% BVL (DM basis) in the Baluchi fattening lambs diet had no significant effect on ADG, whereas feeding 15% BVL (DM basis) decreased BWG (*p* < 0.0001). Feeding BVL15 led to a higher (*p* ≤ 0.05) FCR; however, there were no differences between BVL7.5 and control.

**TABLE 3:** DM intake, body weight (BW), average daily gain (ADG) and feed conversion ratio (FCR) of lambs^a^

	Experimental diets^b^				
Measurement	Control	BVL7.5	BVL15	SEM^c^	*p*‐value
Initial BW (kg)	30.17	30.78	30.24	0.4088	0.5215
Final BW (kg)	43.40^a^	44.10^a^	40.77^b^	0.3955	0.0001
BW gain (kg)	13.22^a^	13.31^a^	10.52^b^	0.2436	0.0001
ADG (g/d)	176.38^a^	177.52^a^	140.38^b^	3.2480	0.0001
Total DM intake (DMI; kg)	65.88^b^	67.26^b^	72.11^a^	0.6579	0.0001
DMI (g/d)	878.49^b^	896.88^b^	961.50^a^	8.7722	0.0001
FCR	4.98^b^	5.06^b^	6.85^a^	0.0997	0.0001

*Note*: Values within a row with different superscripts differ significantly (*p* < 0.05).

^a^ Values are LSM.

^b^ Control, BVL7.5, and BVL15 contained 0%, 7.5% and 15% BVL (DM basis), respectively.

^c^ SEM = pooled standard error of the mean.

### 3.3 Lambs meat composition and colour stability

No differences were found in the chemical composition of LD muscle in lambs fed BVL and control diets (Table 4). The pH value at 24 h in LD muscle was significantly higher in lambs fed BLV, compared to control (*p* < 0.0104). Colour assessment of raw LD muscle (Table 4) showed that yellowness (b* value) and hue angle (H*) were not affected by diet (*p* > 0.05). In contrast, redness (a*) and chroma (C*) were enhanced in BLV‐containing groups (*p* ≤ 0.05). However, lightness (L*) meat in BVL7.5 and BVL15 was significantly lower, compared with the control diet (*p* ≤ 0.05). The shear force index was significantly decreased by adding BVL to the diet (*p* ≤ 0.05), but drip loss and cooking loss of LD muscle were not affected (*p* > 0.05).

**TABLE 4:** Meat quality in longissimus dorsi muscle of lambs fed experimental diets^a^

	Experimental diets^b^				
Measurement	Control	BVL7.5	BVL15	SEM^c^	*p*‐value
DM (%)	25.93	25.95	26.08	0.1107	0.6027
CP (%)	55.46	55.25	54.40	0.6755	0.5366
Ether extract (%)	21.26	21.21	21.24	0.0379	0.6942
Total ash (%)	1.30	1.30	1.29	0.2605	0.8725
pH 24 h	5.68^b^	5.73^a^	5.73^a^	0.0079	0.0104
Lightness (L*)	50.11^a^	47.21^b^	48.41^b^	0.2865	0.0011
Redness (a*)	13.05^b^	14.40^a^	14.20^a^	0.2054	0.0072
Yellowness (b*)	11.15	11.39	11.03	0.2775	0.6755
Hue angle (H*)	40.50	38.33	37.85	1.0310	0.2345
Chroma (C*)	17.17^b^	18.36^a^	17.99^a^	0.1213	0.0012
Drip loss (%)	3.38	3.44	3.36	0.1826	0.9541
Cooking loss (%)	32.28	31.47	32.92	0.6251	0.3306
Shear force (kg)	1.10^a^	1.03^b^	1.03^b^	0.0092	0.0036

*Note*: Values within a row with different superscripts differ significantly (*p* < 0.05).

^a^ Values are LSM.

^b^ Control, BVL7.5 and BVL15 contained 0%, 7.5%, and 15% BVL (DM basis), respectively.

^c^ SEM = pooled standard error of the mean.

### 3.4 Antioxidant indicators in plasma, muscle and liver

**FIGURE 1:**
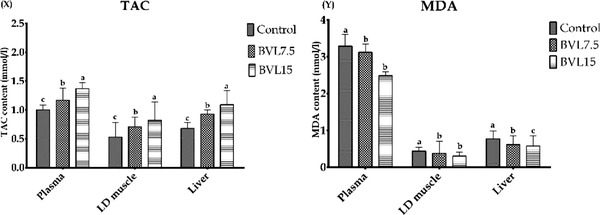
Effect of diets on the (a) total antioxidant capacity (TAC) and (b) malondialdehyde (MDA) values of plasma, *longissimus dorsi* (LD) muscle, and liver of lambs.^a,b,c^ within different superscripts indicate significant differences between dietary treatments (*p* ≤ 0.05). Values are means, with SD represented by vertical bars. BLV7.5, diet containing 7.5% *Berberis vulgaris* leaf (BVL); BVL15, diet containing 15% BVL

TAC in plasma, LD muscle and liver of lambs was significantly increased with the increasing BVL inclusion in diets (*p* ≤ 0.05; Figure 1a). Feeding BVL15 decreased (*p* ≤ 0.05) the MDA concentration of plasma, LD muscle and liver (Figure 1b).

### 3.5 Serum biochemical indicators

Albumin, total protein, total cholesterol, triglyceride, high‐density lipoprotein (HDL), low‐density lipoprotein (LDL), very LDL, blood urea nitrogen (BUN), alkaline phosphatase, glutamic oxaloacetic transaminase and lactate dehydrogenase were not affected by diets (*p* > 0.05; Table 5). Substitution of 15% BVL with alfalfa hay in the diet decreased (*p* ≤ 0.05) blood glucose concentration. Also, glutamic pyruvic transaminase (GPT) increased with the replacement of BVL15 in the diet, but no significant difference was observed between the control diet and BVL7.5 (*p* > 0.05).

**TABLE 5:** Serum biochemical indicators of lambs fed experimental diets^a^

	Experimental diets^b^				
Item	Control	BVL7.5	BVL15	SEM^c^	*p*‐value
Albumin (g/dl)	3.81	3.81	3.79	0.0617	0.9804
Glucose (mg/dl)	77.06^a^	74.84^a^	65.84^b^	2.8233	0.0273
Total protein (g/dl)	6.34	6.49	6.60	0.1259	0.3713
Total cholesterol (mg/dl)	48.26	54.05	53.34	4.0659	0.5579
Triglyceride (mg/dl)	25.06	24.58	24.35	1.2666	0.9230
HDL (mg/dl)	28.41	30.61	28.73	0.9389	0.2291
LDL (mg/dl)	27.84	32.90	33.71	3.8357	0.5151
VLDL (mg/dl)	6.56	5.53	4.89	0.6034	0.1706
BUN (mg/dl)	12.84	13.13	13.47	0.4185	0.5715
ALP (U/L)	69.33	70.60	70.43	2.6225	0.9337
GOT (U/L)	208.37	206.08	199.14	5.2216	0.4449
GPT (U/L)	69.75^b^	70.67^a^, ^b^	75.27^a^, ^b^	1.4959	0.0390
LDH (U/L)	551.44	563.56	623.44	30.4825	0.2294

*Note*: Values within a row with different superscripts differ significantly (*p* < 0.05).

Abbreviations: ALP, alkaline phosphatase; BUN, blood urea nitrogen; GOT, glutamic oxaloacetic transaminase; GPT, glutamic pyruvic transaminase; HDL, high‐density lipoprotein; LDH, lactate dehydrogenase; LDL, low‐density lipoprotein; VLDL, very‐low‐density lipoprotein.

^a^ Values are LSM.

^b^ Control, BVL7.5 and BVL15 contained 0%, 7.5%, and 15% BVL (DM basis), respectively.

^c^ SEM = pooled standard error of the mean.

## 4 DISCUSSION

### 4.1 Chemical composition, phenolic compounds and total alkaloids of BVL

These findings were in agreement with those reported by Modaresi et al. (2016). Likewise, the total alkaloid content of BVL in the current study (1.18 mg/g) was similar to that reported by Ghavipanje et al. (2016). The majority of BVL tannins were hydrolysable, a finding that is supported by previous reports (Ghavipanje et al., 2016; Modaresi et al., 2016). Mazandarani et al. (2013) reported that total phenol, total flavonoid and anthocyanin levels of BVL were 29.4, 59.9 and 11.34 mg CGE/g, respectively. Končić et al. (2010) revealed that the content of total phenol and total flavonoids in different parts of *B vulgaris* ranged between 10.35–52.54 mg GAE/g and 0.24– 4.23 mg QuE/g, respectively. The quantity and chemical compositions depend on the genetic diversity, environmental and growth stage of the plant (Qadir et al., 2009). Hence, the variation in the chemical composition of BVL may be due to differences in cultivar, growing conditions, variety and different processing and analytical methods (Taher‐Maddah et al., 2012).

### 4.2 DMI and growth performance

Few studies have been conducted to examine the nutritive value of BVL in animal nutrition. However, in disagreement with our results, Ghavipanje et al. (2016) observed no differences in total BWG of lactating goats fed 0%, 17% and 34% DM BVL for 42 days, whereas the DMI of goats linearly increased with the increasing BVL proportion in the diets. Consistent with the present results, it is well‐documented that the partial replacement of conventional forages with roughages containing natural antioxidants in livestock diets led to enhanced DMI (Emami et al., 2015; Hukerdi et al., 2019; Huyen et al., 2012). The effects of using unconventional forages on livestock performance are associated with their nutritive value, ruminal digestibility and anti‐nutritional factors (Guil‐Guerrero et al., 2016); in this regard, the nutritional properties of BVL besides low tannin content, especially condensed tannin, compared to pistachio by‐products and pomegranate peel, led to the absence of negative effects on lambs DMI BVL7.5. In contrast, BVL15 caused a significant decrease in FBW, whereas BVL7.5 did not influence the FCR, FBW and total BWG. Although the tannin levels of BVL were low, it had an adverse effect on the performance of lambs. About this, Shahabi and Chashnidel (2014) reported that consumption of flavonoids at low levels due to antimicrobial activity affects microbial balance. In addition, microbial degradation of the flavonoids in the rumen could play an alternative role as a carbon source for microbial activity (Smith et al., 2005). Therefore, high levels of flavonoids in BVL15 due to long‐term exposure (84 days) may decrease the digestion of nutrients and negatively affects lamb's growth performance. Also, blood glucose reduction in lambs fed with BVL15 can be another possible reason for decreasing FBW, BWG and ADG and increasing FCR. Nevertheless, more research are needed to address these effects.

### 4.3 Lambs meat composition and colour stability

The chemical composition of lamb's meat was in line with literature in which animals were fed diets rich in natural antioxidants (Chikwanha et al., 2019; Cohen‐Zinder et al., 2017; Deng et al., 2018; Francisco et al., 2018). The LD muscle of lambs fed BVL had higher pH than those from control. pH is one of the important factors affecting meat tenderness (Devine et al., 1993). The current pH values were within the normal range (5.5 to 5.8) for lambs (Yagoubi et al., 2018). In this experiment, the glycogen content of LD muscle was not measured, but it is possible that lower blood glucose in BVL‐fed lambs could reduce the storage of glycogen in the tissue and thus prevent the degradation of glycogen, glucose and glucose 6‐phosphate to lactate and increase pH, compared to the control group. Also, previous studies show that the use of a mixture of medicinal plant extracts (A. Jang et al., 2008) and gallic acid (K. H. Lee et al., 2012) in broiler chickens and *Pangium edule* extract (containing tannin) in lamb (Patriani et al., 2020) has increased the pH of the meat, compared to the control group. Probably, the increase in phenolic content in the meat of lambs fed with BVL has caused an increase in the pH of the meat (K. H. Lee et al., 2012). Meat colour has been reported as the most important factor when consumers assess meat quality since they relate colour to freshness (Velasco & Williams, 2011). In the present study, the substitution of alfalfa hay with BVL enhanced meat colour and tenderness. The effects of different natural antioxidants on the colour stability of meat are published in the literature (Emami et al., 2015; Hukerdi et al., 2019; Morrissey et al., 1998). Emami et al. (2015) have shown that the use of phenolic compounds did not affect colour stability of raw *longgissimus lumborum* (LL) muscle but significantly increase a* values of LL muscle in Mahabadi goat kids. The redness values observed in the current study (13.02–14.20) exceed the threshold value of ≥ 9.5, which consumers consider acceptable lamb meat colour (Khliji et al., 2010). Several statements that we made were more ambiguous than intended, and we have adjusted the text to be clearer. Although the correlation between myoglobin content and the redness value of meat has been well‐documented (Camacho et al., 2017). Calnan et al. (2016) also reported that the lower myoglobin oxidation is accompanied with higher redness values. In agreement with the results of present study, the inclusion of pomegranate pomace extract has increased redness (a*) and decreased lightness (L*) of the meat (Munir et al., 2019). The lower lightness (L*) and higher redness (a*) in BVL‐fed lambs, compared to the lambs fed the control diet (Table 4), might be attributed to decreased myoglobin oxidation. Several authors have studied the effect of different antioxidants on the colour stability of meat and meat products (Higgins et al., 1998; B. J. Lee et al., 1998) and have reported that pigment oxidation led to lower a* values. H. Zhang et al. (2016) showed the oxidation of pigments can accelerate lipid oxidation, moreover, the production of free fatty acids during lipid oxidation can also oxidise the iron atoms as well as denature the myoglobin molecules and affect meat colour. An increase in the concentration of metmyoglobin and a decrease in the concentration of myoglobin plays a major role in the loss of redness in the meat during chill storage (Adeyemi et al., 2016). Therefore, lower auto‐oxidation through the use of antioxidants, including BVL may decrease the extent of colour degradation in meat. The results of Luciano et al. (2009) show that adding tannins to sheep diets can improve meat colour stability. The increase of a* in lambs fed BVL could be attributed to the antioxidant effect of the polyphenols in the BV, which prevented oxidative deterioration. Therefore, the results of the current experiment confirm the positive effect of BVL on the meat colour stability of lambs.

### 4.4 Antioxidant indicators in plasma, muscle and liver

These findings are in accordance with that presented by Ghavipanje et al. (2016) who reported that the inclusion of 34% BVL (DM basis) in the replacement of alfalfa hay increases the TAC of lactating crossbred goats. In addition, the free radical scavenging activity of BVL has been proved by Motalleb et al. (2005). Similarly, Taheri et al. (2012) have reported that barberry root extract mitigates oxidative stress in rats. The antioxidant activity of BVL compounds such as flavonoids, anthocyanins, vitamin C and isoquinoline alkaloid (i.e., berberine) has been also well‐documented (Brunetton, 1999; Karimov, 1993; Kumar et al., 2015). Generally, a higher intake of natural antioxidants results in the transfer of these molecules in animal tissues with a resultant increase in TAC (Descalzo & Sancho, 2008). Hence, enhancement of plasma, muscle and liver TAC of fattening lambs fed BVL‐containing diets were clearly attributed to transmission of some BVL polyphenols and alkaloids, like berberine and oxyacanthine, to blood and subsequently muscle.

The reaction of TBA with MDA is widely used to measure the rate of muscle fat oxidation (Descalzo & Sancho, 2008; Descalzo et al., 2005). In this regard, the reduction of MDA in blood and LD muscle is another confirmation of the favourable effect of BVL feeding on the meat quality of lambs. The beneficial effects of natural antioxidants‐rich feed staffs such as pomegranate by‐products (Emami et al., 2015; Kotsampasi et al., 2014), rosemary and artemisia (Aouadi et al., 2014), olive leaves (Hukerdi et al., 2019), *Moringa oleifera* (Cohen‐Zinder et al., 2017), tomato pomace (Valenti et al., 2018) and date palm (Sharifi et al., 2015) on antioxidant activity of livestock have been previously confirmed.

### 4.5 Serum biochemical indicators

Information on the effect of BVL in the serum biochemical parameters of ruminants is scarce. However, in agreement with the results of the current study, using 17 and 34 g/kg DM of BVL in lactation dairy goat diets significantly reduces glucose concentration, whereas triglyceride, total cholesterol, HDL, LDL and BUN were not affected (Ghavipanje et al., 2016). Besides, the glucose‐lowering effect of BVL has been proved through in vivo studies using rodent models (Chatuphonprasert et al., 2014; Q. Zhang et al., 2011; Zhou et al., 2009). Ko et al. (2005) reported that berberine (the main alkaloid of BVL) enhances insulin sensitivity by activating protein kinase B and stimulates the glucose uptake by increasing GLUT1 activity and decreasing blood glucose concentration. Furthermore, BVL alkaloids may also decrease glucose transport through the intestinal epithelium to some extent (Pan et al., 2003) and may be another confirmation of the observed glucose‐lowering effect on fattening lambs. In addition, aminotransferases (such as GPT) are often common indicators of cell dysfunction (liver and kidney cells). These enzymes are found in small amounts in plasma, their high amounts are a sign of a disease or malfunction of the liver (Rosenthal, 1997). In this study, it was found that replacing BVL up to 15% of the DM in the diet does not have an adverse effect on the liver function of fattening lambs.

## 5 CONCLUSION

The current experiment tested the hypothesis that alfalfa can be 7.5% replaced by BVL (as a by‐product rich in natural antioxidants) in Baluchi lamb's diet. The findings indicated that feeding BVL to lambs had no adverse effects on DMI and ADG while enhancing the colour stability of meat. Likewise, the TAC of plasma, liver and LD muscle was elevated by BVL substitution in the diet, whereas the MDA content of these tissues was decreased, compared to control, indicating the improvement of the antioxidant status of BVL‐fed lambs. Overall, BVL can be fed to fattening lambs up to 7.5% of the diet (DM basis) as a natural antioxidant by‐product.

## AUTHOR CONTRIBUTIONS

*Conceptualisation, project administration, data curation, formal analysis, funding acquisition, investigation, methodology, farm sampling, software, visualisation, writing–original draft; writing–review and editing*: Seyed Morteza Vaghar Seyedin. *Methodology, supervision*: Mohsen Mojtahedi. *Data curation, formal analysis, software, supervision*: Seyed Homayoun Farhangfar. *Writing–original draft, writing–review and editing*: Navid Ghavipanje.

## CONFLICT OF INTEREST

The authors declare no conflict of interest.

### ETHICS STATEMENT

The authors confirm that the ethical policies of the journal, as noted on the journal's author guidelines page, have been adhered to.

### PEER REVIEW

The peer review history for this article is available at https://publons.com/publon/10.1002/vms3.934.

## Data Availability

The data that support the findings of this study are available from the corresponding author, (S.M. Vaghar Seyedin) upon reasonable request.

## References

[vms3934-bib-0001] Adeyemi, K. D. , Shittu, R. M. , Sabow, A. B. , Abubakar, A. A. , Karim, R. , Karsani, S. A. , & Sazili, A. Q. (2016). Comparison of myofibrillar protein degradation, antioxidant profile, fatty acids, metmyoglobin reducing activity, physicochemical properties and sensory attributes of gluteus medius and infraspinatus muscles in goats. International Journal of Animal Science and Technology, 58(1), 23. 10.1186/s40781-016-0105-5 PMC4908769 27307997

[vms3934-bib-0002] Ahsani, M. R. , Mohammadabadi, M. R. , & Shamsaddini, M. B. (2010). *Clostridium perfringens* isolate typing by multiplex PCR. Journal of Venomous Animals and Toxins, 16, 573–578.

[vms3934-bib-0003] Akbulut, M. , Çalişir, S. , Marakoğlu, T. , & Coklar, H. (2009). Some physicomechanical and nutritional properties of barberry (*Berberis vulgaris* L.) fruits. International Journal of Food Process Engineering, 32(4), 497–511.

[vms3934-bib-0004] Alemardan, A. , Asadi, W. , Rezaei, M. , Tabrizi, L. , & Mohammadi, S. (2013). Cultivation of Iranian seedless barberry (*Berberis integerrima* ‘Bidaneh’): A medicinal shrub. Industrial Crops and Products, 50, 276–287.

[vms3934-bib-0005] AOAC . (2000). Official methods of analysis of AOAC International. AOAC International.

[vms3934-bib-0006] Aouadi, D. , Luciano, G. , Vasta, V. , Nasri, S. , Brogna, D. M. , Abidi, S. , & Salem, H. B. (2014). The antioxidant status and oxidative stability of muscle from lambs receiving oral administration of Artemisia herba alba and *Rosmarinus officinalis* essential oils. Meat Science, 97(2), 237–243. 24583334 10.1016/j.meatsci.2014.02.005

[vms3934-bib-0007] Benzie, I. F. , & Strain, J. J. (1996). The ferric reducing ability of plasma (FRAP) as a measure of “antioxidant power”: The FRAP assay. Analytical Biochemistry, 239(1), 70–76. 8660627 10.1006/abio.1996.0292

[vms3934-bib-0008] Bhatt, J. P. (2016). The effects of *Berberis vulgaris* on bacterial inhibition and the p53 gene in cancerous *Caenorhabditis elegans* . Cancer Research, 76(14), 3525.

[vms3934-bib-0009] Brunetton, J. (1999). Pharmacognosy, phytochemistry, medicinal plants. Lavoisier Publishing.

[vms3934-bib-0010] Camacho, A. , Torres, A. , Capote, J. , Mata, J. , Viera, J. , Bermejo, L. A. , & Argüello, A. (2017). Meat quality of lambs (hair and wool) slaughtered at different live weights. Journal of Applied Animal Research, 45(1), 400–408.

[vms3934-bib-0011] Calnan, H. , Jacob, R. H. , Pethick, D. W. , & Gardner, G. E. (2016). Production factors influence fresh lamb longissimus colour more than muscle traits such as myoglobin concentration and pH. Meat Science, 119, 41–50. 27129082 10.1016/j.meatsci.2016.04.009

[vms3934-bib-0012] Camo, J. , Beltrán, J. A. , & Roncalés, P. (2008). Extension of the display life of lamb with an antioxidant active packaging. Meat Science, 80(4), 1086–1091. 22063841 10.1016/j.meatsci.2008.04.031

[vms3934-bib-0013] Ceccarelli, I. , Bioletti, L. , Peparini, S. , Solomita, E. , Ricci, C. , Casini, I. , Miceli, E. , & Aloisi, A. M. (2021). Estrogens and phytoestrogens in body functions. Neuroscience & Biobehavioral Reviews, 132, 648. 34890602 10.1016/j.neubiorev.2021.12.007

[vms3934-bib-0014] Chatuphonprasert, W. , Lao‐Ong, T. , & Jarukamjorn, K. (2014). Improvement of superoxide dismutase and catalase in streptozotocin–nicotinamide‐induced type 2‐diabetes in mice by berberine and glibenclamide. Pharmaceutical Biology, 52(4), 419–427. 10.3109/13880209.2013.839714 24188560

[vms3934-bib-0015] Chikwanha, O. C. , Muchenje, V. , Nolte, J. E. , Dugan, M. E. , & Mapiye, C. (2019). Grape pomace (*Vitis vinifera* L. cv. Pinotage) supplementation in lamb diets: Effects on growth performance, carcass and meat quality. Meat Science, 147, 6–12. 30172086 10.1016/j.meatsci.2018.08.017

[vms3934-bib-0017] Cohen‐Zinder, M. , Orlov, A. , Trofimyuk, O. , Agmon, R. , Kabiya, R. , Shor‐Shimoni, E. , & Miron, J. (2017). Dietary supplementation of *Moringa oleifera* silage increases meat tenderness of Assaf lambs. Small Ruminant Research, 151, 110–116.

[vms3934-bib-0018] Colomer‐Rocher, F. , Morand‐Fehr, P. , & Kirton, A. (1987). Standard methods and procedures for goat carcass evaluation, jointing and tissue separation. Livestock Production Science, 17, 149–159.

[vms3934-bib-0016] Commission Internationale de I'Eclairage (CIE) . (1986). Colorimetry. 2nd edition, Publication CIE no. 15.2. Commission Internationale de I'Eclairage, Vienna.

[vms3934-bib-0019] Coronado, S. A. , Trout, G. R. , Dunshea, F. R. , & Shah, N. P. (2002). Antioxidant effects of rosemary extract and whey powder on the oxidative stability of wiener sausages during 10 months frozen storage. Meat Science, 62(2), 217–224. 22061414 10.1016/s0309-1740(01)00249-2

[vms3934-bib-0020] da Fonseca, M. D. C. P. , & Salay, E. (2008). Beef, chicken and pork consumption and consumer safety and nutritional concerns in the City of Campinas, Brazil. Food Control, 19(11), 1051–1058.

[vms3934-bib-0021] Deng, K. , Fan, Y. , Ma, T. , Wang, Z. , TanTai, W. , Nie, H. , & Wang, F. (2018). Carcass traits, meat quality, antioxidant status and antioxidant gene expression in muscle and liver of Hu lambs fed perilla seed. Journa of Animal Physiology and Animal Nutrition, 102(2), e828–e837. 10.1111/jpn.12841 29119654

[vms3934-bib-0022] Descalzo, A. , Insani, E. , Biolatto, A. , Sancho, A. , Garcia, P. , Pensel, N. , & Josifovich, J. (2005). Influence of pasture or grain‐based diets supplemented with vitamin E on antioxidant/oxidative balance of Argentine beef. Meat Science, 70(1), 35–44. 22063278 10.1016/j.meatsci.2004.11.018

[vms3934-bib-0023] Descalzo, A. , & Sancho, A. (2008). A review of natural antioxidants and their effects on oxidative status, odor and quality of fresh beef produced in Argentina. Meat Science, 79(3), 423–436. 22062902 10.1016/j.meatsci.2007.12.006

[vms3934-bib-0024] Devine, C. , Graafhuis, A. , Muir, P. , & Chrystall, B. (1993). The effect of growth rate and ultimate pH on meat quality of lambs. Meat Science, 35(1), 63–77. 22060837 10.1016/0309-1740(93)90070-X

[vms3934-bib-0025] Elmore, J. S. , Cooper, S. L. , Enser, M. , Mottram, D. S. , Sinclair, L. A. , Wilkinson, R. G. , & Wood, J. D. (2005). Dietary manipulation of fatty acid composition in lamb meat and its effect on the volatile aroma compounds of grilled lamb. Meat Science, 69(2), 233–242. 22062813 10.1016/j.meatsci.2004.07.002

[vms3934-bib-0026] Emami, A. , Nasri, M. F. , Ganjkhanlou, M. , Zali, A. , & Rashidi, L. (2015). Effects of dietary pomegranate seed pulp on oxidative stability of kid meat. Meat Science, 104, 14–19. 25681560 10.1016/j.meatsci.2015.01.016

[vms3934-bib-0027] Esterbauer, H. , & Cheeseman, K. H. (1990). Determination of aldehydic lipid peroxidation products: malonaldehyde and 4‐hydroxynonenal. Methods in Enzymology, 186, 407–421. 2233308 10.1016/0076-6879(90)86134-h

[vms3934-bib-0028] Francisco, A. , Alves, S. , Portugal, P. , Dentinho, M. , Jerónimo, E. , Sengo, S. , & Alfaia, C. (2018). Effects of dietary inclusion of citrus pulp and rockrose soft stems and leaves on lamb meat quality and fatty acid composition. Animal, 12(4), 872–881. 28988557 10.1017/S1751731117002269

[vms3934-bib-0029] Ghavipanje, N. , Nasri, M. F. , Farhangfar, H. , & Modaresi, J. (2016). *In situ*, *in vitro* and *in vivo* nutritive value assessment of Barberry leaf as a roughage for goat feeding. Small Ruminant Research, 141, 94–98.

[vms3934-bib-0030] Grunert, K. G. (2006). Future trends and consumer lifestyles with regard to meat consumption. Meat Science, 74(1), 149–160. 22062724 10.1016/j.meatsci.2006.04.016

[vms3934-bib-0031] Guil‐Guerrero, J. , Ramos, L. , Moreno, C. , Zúñiga‐Paredes, J. , Carlosama‐Yépez, M. , & Ruales, P. (2016). Plant‐food by‐products to improve farm‐animal health. Animal Feed Science and Technology, 220, 121–135.

[vms3934-bib-0032] Higgins, F. , Kerry, J. , Buckley, D. , & Morrissey, P. (1998). Effect of dietary α‐tocopheryl acetate supplementation on α‐tocopherol distribution in raw turkey muscles and its effect on the storage stability of cooked turkey meat. Meat Science, 50(3), 373–383. 22061156 10.1016/s0309-1740(98)00045-x

[vms3934-bib-0033] Hopkins, D. , & Thompson, J. (2001). The relationship between tenderness, proteolysis, muscle contraction and dissociation of actomyosin. Meat Science, 57(1), 1–12. 22061160 10.1016/s0309-1740(00)00065-6

[vms3934-bib-0034] Hukerdi, Y. J. , Nasri, M. F. , Rashidi, L. , Ganjkhanlou, M. , & Emami, A. (2019). Effects of dietary olive leaves on performance, carcass traits, meat stability and antioxidant status of fattening Mahabadi male kids. Meat Science, 153, 2–8. 30856549 10.1016/j.meatsci.2019.03.002

[vms3934-bib-0035] Huyen, V. , Phan, D. , Thang, P. , Ky, P. , Hoa, N. , & Ostenson, C. (2012). Antidiabetic effects of add‐on *Gynostemma pentaphyllum* extract therapy with sulfonylureas in type 2 diabetic patients. Evidence‐Based Complementary and Alternative Medicine, 2012, 452313. 23125867 10.1155/2012/452313 PMC3484409

[vms3934-bib-0036] Imanshahidi, M. , & Hosseinzadeh, H. (2008). Pharmacological and therapeutic effects of *Berberis vulgaris* and its active constituent, berberine. Phytotherapy Research, 22(8), 999–1012. 18618524 10.1002/ptr.2399

[vms3934-bib-0037] Jang, A. , Liu, X. D. , Shin, M. H. , Lee, B. D. , Lee, S. K. , Lee, J. H. , & Jo, C. (2008). Antioxidative potential of raw breast meat from broiler chicks fed a dietary medicinal herb extract mix. Poultry Science, 87(11), 2382–2389. 10.3382/ps.2007-00506 18931191

[vms3934-bib-0038] Jang, M. H. , Kim, H. Y. , Kang, K. S. , Yokozawa, T. , & Park, J. H. (2009). Hydroxyl radical scavenging activities of isoquinoline alkaloids isolated from *Coptis chinensis* . Archives of Pharmacal Research, 32(3), 341–345. 19387576 10.1007/s12272-009-1305-z

[vms3934-bib-0039] Karabagias, I. , Badeka, A. , & Kontominas, M. (2011). Shelf life extension of lamb meat using thyme or oregano essential oils and modified atmosphere packaging. Meat Science, 88(1), 109–116. 21211912 10.1016/j.meatsci.2010.12.010

[vms3934-bib-0040] Karimov, A. (1993). Berberis alkaloids. Chemistry of Natural Compounds, 29(4), 415–438.

[vms3934-bib-0041] Khliji, S. , Van de Ven, R. , Lamb, T. , Lanza, M. , & Hopkins, D. (2010). Relationship between consumer ranking of lamb colour and objective measures of colour. Meat Science, 85(2), 224–229. 20374889 10.1016/j.meatsci.2010.01.002

[vms3934-bib-0042] Ko, B. S. , Choi, S. B. , Park, S. K. , Jang, J. S. , Kim, Y. E. , & Park, S. (2005). Insulin sensitizing and insulinotropic action of berberine from *Cortidis rhizoma* . Biological & Pharmaceutical Bulletin, 28(8), 1431–1437. 16079488 10.1248/bpb.28.1431

[vms3934-bib-0043] Končić, M. Z. , Kremer, D. , Karlović, K. , & Kosalec, I. (2010). Evaluation of antioxidant activities and phenolic content of *Berberis vulgaris* L. and *Berberis croatica* Horvat. Food and Chemical Toxicology, 48(8), 2176–2180. 20488218 10.1016/j.fct.2010.05.025

[vms3934-bib-0044] Kotsampasi, B. , Christodoulou, V. , Zotos, A. , Liakopoulou‐Kyriakides, M. , Goulas, P. , Petrotos, K. , & Bampidis, V. (2014). Effects of dietary pomegranate byproduct silage supplementation on performance, carcass characteristics and meat quality of growing lambs. Animal Feed Science and Technology, 197, 92–102.

[vms3934-bib-0045] Krishtafovich, V. , Krishtafovich, D. , Surzhanskaya, I. , Marakova, A. , & Vorobieva, D. (2016). The value of the lamb meat in human nutrition. *International* Food Research Journal, 23(6), 2540.

[vms3934-bib-0046] Kumar, A. , Chopra, K. , Mukherjee, M. , Pottabathini, R. , & Dhull, D. K. (2015). Current knowledge and pharmacological profile of berberine: An update. European Journal of Pharmacology, 761, 288–297. 26092760 10.1016/j.ejphar.2015.05.068

[vms3934-bib-0047] Laamech, J. , El‐Hilaly, J. , Fetoui, H. , Chtourou, Y. , Gouitaa, H. , Tahraoui, A. , & Lyoussi, B. (2017). *Berberis vulgaris* L. effects on oxidative stress and liver injury in lead‐intoxicated mice. Journal of Complementary and Integrative Medicine, 14(1), 20170301. 10.1515/jcim-2015-0079 28195545

[vms3934-bib-0048] Lee, B. J. , Hendricks, D. G. , & Cornforth, D. P. (1998). Antioxidant effects of carnosine and phytic acid in a model beef system. International Journal of Food Science, 63(3), 394–398.

[vms3934-bib-0049] Lee, K. H. , Jung, S. , Kim, H. J. , Kim, I. S. , Lee, J. H. , & Jo, C. (2012). Effect of dietary supplementation of the combination of gallic and linoleic acid in thigh meat of broilers. Asian‐Australasian Journal of Animal Sciences, 25(11), 1641. 25049528 10.5713/ajas.2012.12260 PMC4093043

[vms3934-bib-0050] Lenahan, M. , O'Brien, S. , Kinsella, K. , Sweeney, T. , & Sheridan, J. (2007). Prevalence and molecular characterization of *Escherichia coli* O157: H7 on Irish lamb carcasses, fleece and in faeces samples. Journal of Applied Microbiology, 103(6), 2401–2409. 18045425 10.1111/j.1365-2672.2007.03476.x

[vms3934-bib-0051] Lokman, N. S. , Sabow, A. B. , Abubakar, A. A. , Adeyemi, K. D. , & Sazili, A. Q. (2017). Comparison of carcass and meat quality in goats subjected to preslaughter head‐only electrical stunning or slaughtered without stunning. CyTA Journal of Food, 15(1), 99–104.

[vms3934-bib-0052] Luciano, G. , Monahan, F. , Vasta, V. , Pennisi, P. , Bella, M. , & Priolo, A. (2009). Lipid and colour stability of meat from lambs fed fresh herbage or concentrate. Meat Science, 82(2), 193–199. 20416762 10.1016/j.meatsci.2009.01.010

[vms3934-bib-0053] Makkar, H. P. , Blümmel, M. , Borowy, N. K. , & Becker, K. (1993). Gravimetric determination of tannins and their correlations with chemical and protein precipitation methods. Journal of the Science of Food and Agriculture, 61(2), 161–165.

[vms3934-bib-0054] Makkar, H. P. , Tran, G. , Heuzé, V. , & Ankers, P. (2014). State‐of‐the‐art on use of insects as animal feed. Animal Feed Science and Technology, 197, 1–33.

[vms3934-bib-0055] Masoudzadeh, S. H. , Mohammadabadi, M. R. , Khezri, A. , Kochuk‐Yashchenko, O. A. , Kucher, D. M. , Babenko, O. I. , Bushtruk, M. V., Tkachenko, S. V., Stavetska, R. V., Klopenko, N. I., Oleshko, V. P., Tkachenko, M. V, & Titarenko, I. V. (2020). Dlk1 gene expression in different tissues of lamb. Iranian Journal of Applied Animal Science, 10(4), 669–677.

[vms3934-bib-0056] Mazandarani, M. , Ghaderi, N. , & Bayat, H. (2013). Investigation of secondary active compounds of medical plant *Berberis vulgaris* L. and its comparison among different part of the plant in south east of Golestan province. Journal of Plant Environmental Physiology, 8(special issue), 59–70.

[vms3934-bib-0057] Meliani, N. , Dib, M. E. A. , Allali, H. , & Tabti, B. (2011). Hypoglycaemic effect of *Berberis vulgaris* L. in normal and streptozotocin‐induced diabetic rats. Asian Pacific Journal of Tropical Biomedicine, 1(6), 468–471. 23569815 10.1016/S2221-1691(11)60102-0 PMC3614224

[vms3934-bib-0058] Modaresi, J. , Valizadeh, R. , Fathinasari, M. , Mousavi, A. , Mesgaran, M. , & Khosravi, F. (2016). Evaluation of nutritive value, phenolic compounds and in vitro digestion characteristics of barberry (*Berberis vulgaris*) foliage. Iranian Journal of Animal Science Research, 8(2), 227–237.

[vms3934-bib-0059] Mohammadabadi, M. , Kord, M. , & Nazari, M. (2018). Studying expression of leptin gene in different tissues of Kermani Sheep using Real Time PCR. Agricultural Biotechnology Journal, 10, 111–23.

[vms3934-bib-0060] Mokhber‐Dezfuli, N. , Saeidnia, S. , Gohari, A. R. , & Kurepaz‐Mahmoodabadi, M. (2014). Phytochemistry and pharmacology of berberis species. Pharmacognosy Reviews, 8(15), 8. 24600191 10.4103/0973-7847.125517 PMC3931204

[vms3934-bib-0061] Morrissey, P. , Sheehy, P. , Galvin, K. , Kerry, J. , & Buckley, D. (1998). Lipid stability in meat and meat products. Meat Science, 49, S73–S86. 22060722

[vms3934-bib-0062] Motalleb, G. , Hanachi, P. , Kua, S. , Fauziah, O. , & Asmah, R. (2005). Evaluation of phenolic content and total antioxidant activity in *Berberis vulgaris* fruit extract. Journal of Biological Sciences, 5(5), 648–653.

[vms3934-bib-0063] Munir, S. , Hu, Y. , Liu, Y. , & Xiong, S. (2019). Enhanced properties of silver carp surimi‐based edible films incorporated with pomegranate peel and grape seed extracts under acidic condition. Food Packaging and Shelf Life, 19, 114–120.

[vms3934-bib-0064] Murugesh, K. S. , Channabasappa Yeligar, V. , Maiti, B. C. , & Kumar Maity, T. (2005). Hepato protective and antioxidant role of *Berberis tinctoria* Lesch leaves on paracetamol induced hepatic damage in rats. Iranian journal of Pharmacology & Therapeutics, 4(1), 64–60.

[vms3934-bib-0065] NRC. National Research council. (2007). Nutrient requirements of small ruminants: Sheep, goats, cervids, and New World camelids. National Academies Press.

[vms3934-bib-0066] Osés, S. M. , Diez, A. M. , Melero, B. , Luning, P. A. , Jaime, I. , & Rovira, J. (2013). Characterization by culture‐dependent and culture‐independent methods of the bacterial population of suckling‐lamb packaged in different atmospheres. Food Microbiology, 36(2), 216–222. 24010600 10.1016/j.fm.2013.05.005

[vms3934-bib-0067] Pan, G. ‐Y. , Huang, Z. ‐J. , Wang, G. ‐J. , Fawcett, J. P. , Liu, X. ‐D. , Zhao, X. ‐C. , & Xie, Y. Y. (2003). The antihyperglycaemic activity of berberine arises from a decrease of glucose absorption. Planta Medica, 69(7), 632–636. 12898419 10.1055/s-2003-41121

[vms3934-bib-0068] Patriani, P. , Hafid, H. , Mirwandhono, E. , Wahyuni, T. H. , Hasanah, U. , Apsari, N. L. , & Ginting, N. (2020). Physical quality characteristics of lamb meat using *Pangium edule* extract at different storage times. IOP Conference Series: Earth and Environmental Science, 454(1), 012056.

[vms3934-bib-0069] Placer, Z. A. , Cushman, L. L. , & Johnson, B. C. (1966). Estimation of product of lipid peroxidation (malonyl dialdehyde) in biochemical systems. Analytical Biochemistry, 16(2), 359–364. 6007581 10.1016/0003-2697(66)90167-9

[vms3934-bib-0070] Qadir, S. A. , Kwon, M. C. , Han, J. G. , Ha, J. H. , Chung, H. S. , Ahn, J. , & Lee, H. Y. (2009). Effect of different extraction protocols on anticancer and antioxidant activities of *Berberis koreana* bark extracts. Journal of Bioscience and Bioengineering, 107(3), 331–338. 19269602 10.1016/j.jbiosc.2008.11.021

[vms3934-bib-0071] Rahimi‐Madiseh, M. , Lorigoini, Z. , Zamani‐Gharaghoshi, H. , & Rafieian‐Kopaei, M. (2017). *Berberis vulgaris*: Specifications and traditional uses. IJBMS, 20(5), 569. 28656092 10.22038/IJBMS.2017.8690 PMC5478785

[vms3934-bib-0072] Rapisarda, P. , Fanella, F. , & Maccarone, E. (2000). Reliability of analytical methods for determining anthocyanins in blood orange juices. Journal of Agricultural and Food Chemistry, 48(6), 2249–2252. 10888531 10.1021/jf991157h

[vms3934-bib-0073] Rosenthal, P. (1997). Assessing liver function and hyperbilirubinemia in the newborn. Clinical Chemistry, 43(1), 228–234. 8990258

[vms3934-bib-0074] Saki, K. , Eftekhari, Z. , Naghdi, N. , & Bahmani, M. (2016). *Berberis vulgaris* as an antihypertensive drug; berbamine and oxycontin antihypertensive active ingredients. Journal of Preventive Epidemiology, 1(2), e18.

[vms3934-bib-0076] Shahabi, H. , & Chashnidel, Y. (2014). The effects of canola oil and oregano essential oil on performance, blood parameters, and chemical carcass compositions of Dalagh fattening lambs. Journal of Ruminant Research, 2(1), 33–50.

[vms3934-bib-0077] Shahsavari, M. , Mohammadabadi, M. , Khezri, A. , Asadi Fozi, M. , Babenko, O. , Kalashnyk, O. , Oleshko, V. , & Tkachenko, S. (2021). Correlation between insulin‐like growth factor 1 gene expression and fennel (*Foeniculum vulgare*) seed powder consumption in muscle of sheep. Animal Biotechnology. Advance online publication. 10.1080/10495398.2021.2000997 34783639

[vms3934-bib-0078] Shamsa, F. , Ahmadiani, A. , & Khosrokhavar, R. (1999). Antihistaminic and anticholinergic activity of barberry fruit (*Berberis vulgaris*) in the guinea‐pig ileum. Journal of Ethnopharmacology, 64(2), 161–166. 10197751 10.1016/s0378-8741(98)00122-6

[vms3934-bib-0079] Shamsa, F. , Monsef, H. , Ghamooshi, R. , & Verdian‐rizi, M. (2008). Spectrophotometric determination of total alkaloids in some Iranian medicinal plants. Thai Journal of Pharmaceutical Sciences, 3*2*, 17–20.

[vms3934-bib-0080] Sharifi, M. , Bashtani, M. , Naserian, A. A. , & Farhangfar, H. (2015). The effect of feeding low quality date palm (*Phoenix dactylifera* L.) on the performance, antioxidant status and ruminal fermentation of mid‐lactating Saanen dairy goats. Small Ruminant Research, 130, 95–100.

[vms3934-bib-0081] Sierra, M. ‐L. , Gonzalez‐Fandos, E. , García‐López, M. ‐L. , Fernandez, M. C. G. , & Prieto, M. (1995). Prevalence of *Salmonella*, *Yersinia*, *Aeromonas*, *Campylobacter*, and cold‐growing *Escherichia coli* on freshly dressed lamb carcasses. Journal of Food Protection, 58(11), 1183–1185. 31137311 10.4315/0362-028X-58.11.1183

[vms3934-bib-0082] Smith, A. H. , Zoetendal, E. , & Mackie, R. I. (2005). Bacterial mechanisms to overcome inhibitory effects of dietary tannins. Microbiology Ecoogy, 50(2), 197–205. 10.1007/s00248-004-0180-x 16222487

[vms3934-bib-0075] Statistical Analysis System (SAS) Institute . (2002). SAS/STAT User’s Guide . Version 8, 6th Edition, SAS Institute, Cary, 112.

[vms3934-bib-0083] Taher‐Maddah, M. , Maheri‐Sis, N. , Salamatdoustnobar, R. , & Ahmadzadeh, A. (2012). Comparing nutritive value of ensiled and dried pomegranate peels for ruminants using in vitro gas production technique. Annals of Applied Biology, 3(4), 1942–1946. PMC4655782 26623290

[vms3934-bib-0084] Taheri, S. , Zarei, A. , Ashtiyani, S. C. , Rezaei, A. , & Zaheiri, S. (2012). Evaluation of the effects of hydroalcoholic extract of *Berberis vulgaris* root on the activity of liver enzymes in male hypercholesterolemic rats. Avicenna Journal of Phytomedicine, 2(3), 153. 25050245 PMC4075670

[vms3934-bib-0085] Valenti, B. , Luciano, G. , Pauselli, M. , Mattioli, S. , Biondi, L. , Priolo, A. , & Lanza, M. (2018). Dried tomato pomace supplementation to reduce lamb concentrate intake: Effects on growth performance and meat quality. Meat Science, 145, 63–70. 29906738 10.1016/j.meatsci.2018.06.009

[vms3934-bib-0086] Van Soest, P. V. , Robertson, J. , & Lewis, B. (1991). Methods for dietary fiber, neutral detergent fiber, and nonstarch polysaccharides in relation to animal nutrition. Journal of Dairy Science, 74(10), 3583–3597. 1660498 10.3168/jds.S0022-0302(91)78551-2

[vms3934-bib-0087] Velasco, V. , & Williams, P. (2011). Improving meat quality through natural antioxidants. Chilean Journal of Agricultural Research, 71(2), 313.

[vms3934-bib-0088] Wu, G. , Liu, X. , Chen, T. , Xu, G. , Wang, W. , Zeng, X. , & Zhang, X. (2015). Elevation‐dependent variations of tree growth and intrinsic water‐use efficiency in Schrenk spruce (*Picea schrenkiana*) in the western Tianshan Mountains, China. Frontiers in Plant Science, 6, 309. 25999973 10.3389/fpls.2015.00309 PMC4422019

[vms3934-bib-0089] Yagoubi, Y. , Joy, M. , Ripoll, G. , Mahouachi, M. , Bertolín, J. , & Atti, N. (2018). Rosemary distillation residues reduce lipid oxidation, increase alpha‐tocopherol content and improve fatty acid profile of lamb meat. Meat Science, 136, 23–29. 29059539 10.1016/j.meatsci.2017.10.007

[vms3934-bib-0090] Yang, N. , Sun, R. ‐B. , Chen, X. ‐L. , Zhen, L. , Ge, C. , Zhao, Y. ‐Q. , & Yu, X. ‐Y. (2017). In vitro assessment of the glucose‐lowering effects of berberrubine‐9‐O‐β‐D‐glucuronide, an active metabolite of berberrubine. Acta Physica Sinica, 38(3), 351. 10.1038/aps.2016.120 PMC5342660 28042874

[vms3934-bib-0091] Zhang, H. , Wu, J. , & Guo, X. (2016). Effects of antimicrobial and antioxidant activities of spice extracts on raw chicken meat quality. LWT–Food Science and Technology, 5(1), 39–48.

[vms3934-bib-0092] Zhang, Q. , Xiao, X. , Feng, K. , Wang, T. , Li, W. , Yuan, T. , & Wang, H. (2011). Berberine moderates glucose and lipid metabolism through multipathway mechanism. Evidence‐Based Complementary Alternative Medicine, 2011, 924851. 20953398 10.1155/2011/924851 PMC2952334

[vms3934-bib-0093] Zhou, J. , Zhou, S. , Tang, J. , Zhang, K. , Guang, L. , Huang, Y. , & Li, D. (2009). Protective effect of berberine on beta cells in streptozotocin‐and high‐carbohydrate/high‐fat diet‐induced diabetic rats. European Journal of Pharmacology, 606(1‐3), 262–268. 19374872 10.1016/j.ejphar.2008.12.056

